# EZH2-TROAP Pathway Promotes Prostate Cancer Progression *Via* TWIST Signals

**DOI:** 10.3389/fonc.2020.592239

**Published:** 2021-02-22

**Authors:** Lu Jin, Yibin Zhou, Guangqiang Chen, Guangcheng Dai, Kai Fu, Dongrong Yang, Jin Zhu

**Affiliations:** ^1^ Department of Urology, Second Affiliated Hospital of Soochow University, Suzhou, China; ^2^ Department of Radiology, Second Affiliated Hospital of Soochow University, Suzhou, China

**Keywords:** prostate cancer, EZH2, TWIST, c-Myc, trophinin associated protein

## Abstract

Trophinin-associated protein (TROAP) has been shown to be overexpressed and promotes tumor progression in some tumors. We performed this study to assess the biological and clinical significance of TROAP in prostate cancer. We downloaded TROAP mRNA expression data from TCGA and GEO databases. We analyzed expressions of TROAP and other genes in prostate cancer tumors at different stages and assessed Gleason scores. We used Celigo image, Transwell, and rescue assays, and flow cytometry detection to assess growth, apoptosis, proliferation, migration, and invasion of the prostate cancer cells. We identified and validated up- and down-stream genes in the TROAP pathway. The mRNA data suggested that TROAP expression was markedly upregulated in prostate cancer compared with its expression in normal tissues, especially in cancers with high stages and Gleason scores. Moreover, a high TROAP expression was associated with poor patient survival. Results of our *in vitro* assay showed that TROAP knockdown inhibited DU145 and PC3 cell proliferation and viability *via* cell apoptosis and S phase cycle arrest. The Transwell assay showed that TROAP knockdown inhibited cell migration and invasion, probably through MMP-9 and E-Cadherin modulation. Overexpression of TWIST partially abrogated the inhibitory effects of TROAP knockdown on prostate cancer cells. Our integrative mechanism dissection revealed that TROAP is in a pathway downstream of EZH2 and that it activates the TWIST/c-Myc pathway to regulate prostate cancer progression. In all, we identified TROAP as a driver of prostate cancer development and progression, providing a novel target for prostate cancer treatments.

## Introduction

Prostate cancer is the most common malignant tumor in men and ranks as the leading cause of cancer-related mortality in most countries of the world (1, 2). Although multiple therapeutic strategies, including surgery, ADT, and chemotherapy, are initially highly effective, progression eventually occurs years later (3, 4). Clarifying the molecular mechanisms of prostate cancer progression is important to improve prostate cancer therapy.

TROAP, also called tastin, interacts with trophinin (5, 6). Previous spatial expression profile studies indicated that TROAP mRNA is highly expressed in some normal tissues and tumors (6–8). According to previous findings, a high TROAP expression predicts poor prognosis or tumor progression in ovarian and breast cancers and in hepatocellular carcinoma (8–10). TROAP has been shown to promote some tumor progressions, such as those of breast and colorectal cancers (9, 11). In prostate cancers, reduced TROAP expression can inhibit cancer cell proliferation and induce a cell cycle arrest (12). However, the mechanisms of TROAP in prostate cancer remain obscure. In this study, we first compared the expression levels of TROAP between prostate cancer tissues and paired normal tissues using data from TCGA and GEO databases. In addition, to explore the functions of TROAP in prostate cancer, we performed gene coexpression analyses with *in vitro* assays in prostate cancer cell lines.

## Materials and Methods

### Microarray Data Sources

We downloaded TROAP mRNA expression and clinical information of patients with prostate cancer from the TCGA database and the public GEO repository. We calculated *P*-values using the Student’s *t*-tests.

### Cell Lines and Culture

The human prostate cancer cell lines DU145 and PC-3 were purchased from the Cell Bank of the Chinese Academy of Science (Shanghai, USA). All cells lines were incubated in DMEM (Hyclone Laboratories; GE Healthcare Life Sciences, Logan, UT, USA) with 10% fetal bovine serum (FBS) and cultured at 37 °C in a humidified atmosphere with 5% CO_2_. Antibiotic solutions of 100 U/ml penicillin G and 100 μg/ml streptomycin were added to the culture medium to prevent bacterial contamination.

### RNA Extraction and qPCR

Total RNA was isolated from DU145 and PC-3 cells using the RNAiso Plus reagent (Takara Bio, Otsu, Japan). Next, 1-*μg* total RNA samples were isolated using a PrimeScript™ RT reagent kit (Takara Bio). qPCRs were performed using Premix Ex Taq™ II (Takara Bio) with a Roche Light Cycler 480 Real-Time PCR system and using *β*-actin as the internal control. The reverse reaction conditions were as follows: 95 °C for 1 min, 40 cycles of 95 °C for 5 s, 60 °C for 30 s, and 75 °C for 30 s. The primer sequences used were the following: TROAP (forward, 5′-GGTCAGGAGAAAAGGGGAGGAAG-3′; reverse, 5′-AGGCGTGCGTTTCTGAGAGC-3′); and *β*-actin (forward, 5′-GTGGACATCCGCAAAGAC-3′; reverse, 5′-AAAGGGTGTAACGCAACTA-3′). We used the 2^-ΔΔCq^ method to calculate the relative TROAP expression levels.

### Lentivirus Infection of Prostate Cancer Cells

We designed a TROAP-shRNA by connecting the shTROAP sequence to the pGreenPuro™ Cloning and Expression Lentivector (SBI System Biosciences, Palo Alto, CA, USA). The sequence of shTROAP was: 5′-AGAACCAAGATCCAAGGAGATCTCGAGATCTCCTTGGATCTTGGTTCT-3′. And, the control-shRNA was 5′-TTCTCCGAACGTGTCACGTCTCGAGACGTGACACGTTCGGAGAA-3′. Lentiviral particles were produced in 93T cells and harvested by ultra-centrifugation. For TROAP downregulation, we used the lentivirus containing shTROAP to infect DU145 and PC-3 cell lines.

### Western Blot Analyses

We extracted whole cell proteins from prostate cancer cells after lysis in RIPA buffer (Beyotime, Haimen, China). Total protein samples were quantified using the Coomassie brilliant blue method. We loaded supernatant samples onto SDS-PAGE gels and separated the proteins before electro-transferring them onto PVDF membranes (Merck Millipore, Darmstadt, Germany). The membranes were further incubated with specific antibodies listed in the **Supplementary Table 1**. After tris buffer solution washes, we incubated specific protein bands with secondary antibody for 1 h and detected the signal using enhanced chemiluminescent assays.

### Cell Growth Assays

We evaluated the effects of TROAP knockdown in prostate cancer cell growth using Celigo image cytometry. Lentivirus-infected DU145 and PC-3 cells were incubated at 37 °C in a 5% humidified atmosphere for 5 days. We used a Celigo Imaging Cytometer (Nexcelom Bioscience, Lawrence, MA, USA) to conduct cell count assays.

### Cell Viability Assays

We used the 3-(4, 5-dimethyl-2-thiazolyl)-2, 5-diphenyl-2h-tetrazolium bromide (MTT) proliferation assay to assess cell viability. After 12-h cultures, lentivirus-infected DU145 and PC-3 cells were placed onto 96-well plates (2,000 cells/well) and incubated for 5 days. Next, MTT and an acidic isopropanol solution were added to every well for 1 h at 37°C. The absorbance at 595 nm was measured in a spectrophotometric plate reader (BioTek Instruments, Winooski, VT, USA).

### Flow Cytometry Detection

We assessed the cell cycle and apoptosis rates of prostate cancer cells using flow cytometric analyses. All infected DU145 and PC-3 cells were inoculated with trypsinization. For the cell cycle analysis, after harvesting and washing them with phosphate-buffered saline (PBS) twice, we incubated the cells in the dark after adding the propidium iodide (PI) and then scanned them in a FACScan (BD Biosciences, Franklin Lakes, NJ, USA). We calculated and compared the percentages of prostate cancer cells in three cycle phases. For the cell apoptosis analyses, after washing the cells in PBS, we cultured apoptotic and necrotic cells for 30 min at 37°C after adding 5 μl of FITC-Annexin V and 10 μl of PI. We calculated stained cells and apoptosis rates using the FACScan (BD Biosciences).

### Cell Invasion and Migration Assay

We used a Transwell assay to detect the invasion and migration abilities of DU145 and PC-3 cells 96 h after transfection. After washing the cells with the BPS, we seeded them into 24-well plates at a density of 1 × 10^5^ cells/well. We added FBS as a chemoattractant to the lower chamber. After 24 h of incubation, we washed the invasive cells located on the bottom membrane surface using PBS, fixed them in 95% ethanol, and stained them with 0.1% crystal violet. We randomly selected three independent visual fields, and determined the number of stained cells under a light microscope (Lecia, Germany).

### Statistical Analysis

We used Student *t* tests to assess associations between different variables with a continuous distribution and Pearson tests to analyze correlations between genes. We considered *P*-values < 0.05 as statistically significant. All statistical analyses were performed using the SPSS 24.0 software package and GraphPad Prism Software 6.0.

## Results

### TROAP Is Over-Expressed in Prostate Cancer Cells

**Figure 1:**
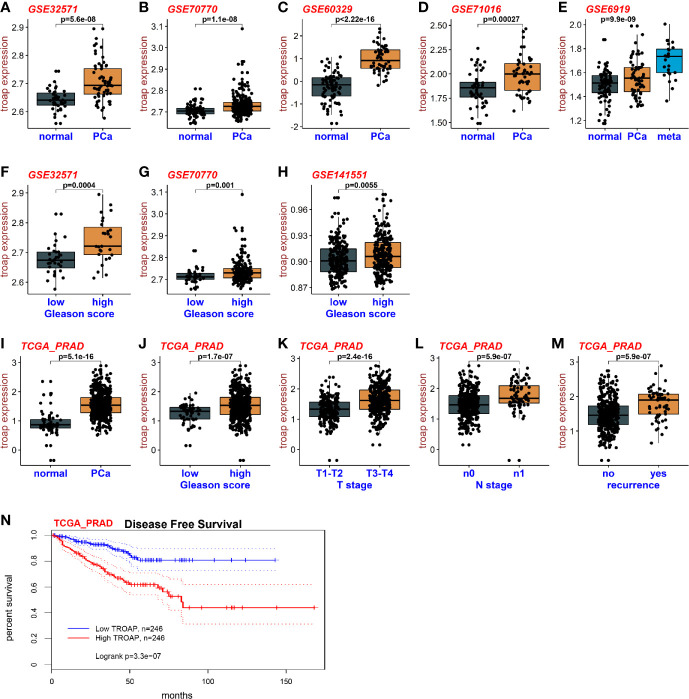
Expression of TROAP in prostate cancer. The expression of TROAP mRNA in prostate cancer tissues was higher than that in normal tissues in different GEO datasets **(A–D)**. TROAP expression was high in prostate cancer with metastasis **(E)**. TROAP expression was high in prostate cancer samples with high Gleason scores **(F–H)**. Results from the PRAD show that TROAP was overexpressed in prostate cancer tissues **(I)**. TROAP expressions were high in prostate cancer with high Gleason scores, T and N stages, and recurrent tumors **(J–M)**. High TROAP expression predicts a poor disease-free survival **(N)**.

To assess the expression levels of TROAP mRNA in prostate cancer and normal tissues, we downloaded RNA sequencing data from the TCGA and GEO databases. Base on the GEO datasets GSE32571, GSE70770, GSE60329 and GSE71016 (**Figures 1A–D**), the results showed that TROAP mRNA is overexpressed in prostate cancer tissues compared with which in normal tissues. The GSE6919 dataset showed similar results with TROAP mRNA being overexpressed in cancer tissues, but the overexpression was especially high in tissues with metastasis (**Figure 1E**). Our results also showed that cancers with high Gleason scores had high TROAP mRNA expression levels (GSE32571, **Figure 1F**; GSE70770, **Figure 1G**; GSE141551, **Figure 1H**). Results from the TCGA prostate cancer (PRAD) database showed similar results with TROAP mRNA being overexpressed in prostate cancer tissues compared with the expression in normal prostate tissues (**Figure 1I**). Data from PRAD also showed that expression of TROAP mRNA was high in prostate cancer with high Gleason scores (**Figure 1J**), high T stages (**Figure 1K**), high N stages (**Figure 1L**) and recurrent tumors (**Figure 1M**). We used information from the PRAD to perform disease free survival analyses and our results show that a high TROAP expression predicted a poor disease free survival rate (**Figure 1N**, we used the mean TROAP expression to set a cutoff value to divide patients into high and low TROAP groups). Our results suggest that TROAP is associated with tumorigenesis and development of prostate cancer.

### TROAP Knockdown Inhibited Prostate Cancer Cell Proliferation and Viability

**Figure 2:**
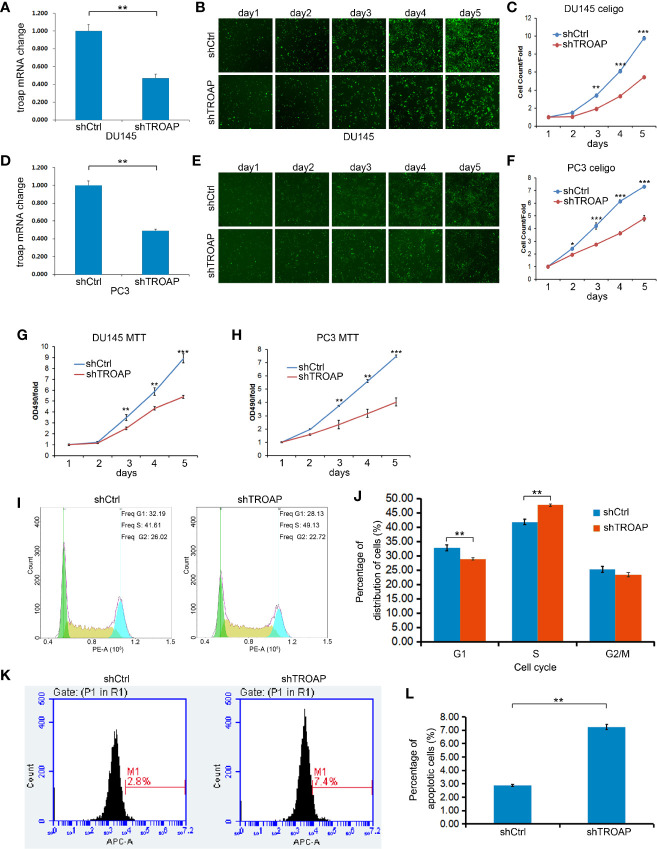
Infection with shTROAP lentivirus inhibited expression of TROAP in DU145 cells **(A)**. TROAP knockdown suppressed DU145 cell proliferation **(B, C)**. Similar results were observed in PC3 cells **(D–F)**. The MTT assay results show that TROAP knockdown inhibited prostate cancer cell (DU145 and PC3) viability **(G, H)**. TROAP knockdown induced S phase arrest of DU145 cells **(I, J)**. TROAP knockdown induced DU145 cell apoptosis **(K, L)**. **P < 0.01, ***P < 0.001.

To investigate the effects of TROAP on DU145 and PC-3 cell proliferation and viability, we performed MTT assays and calculated cell numbers using a Celigo cell imaging analyzer. TROAP was knocked down by 55.71% (in DU145 cells) and 54.13% (in PC3 cells) in cells infected with shTROAP lentivirus, when compared to the expressions in the same cells infected with shCtrl lentivirus (**Figures 2A, D**, *P <* 0.01). In DU145 (**Figures 2B, C**) and PC3 (**Figures 2E, F**) cells, TROAP knockdown significantly inhibited cell proliferation on the 3^rd^, 4^th^ and 5^th^ days after infection. The MTT assay results also showed that TROAP knockdown inhibited cell viability by 35.43% on the 3^rd^ day (*P <* 0.01), 33.82% on the 4^th^ day (*P <* 0.01), and 45.09% on the 5^th^ day (*P <* 0.001) in DU145 cells, and by 34.63% on the 3^rd^ day (*P <* 0.01), 40.52% on the 4^th^ day (*P <* 0.01), and 43.66% on the 5^th^ (*P <* 0.001) in PC3 cells, compared with the viabilities of the shCtrl cells on at the same timepoints (**Figures 2G, H**).

### TROAP Knockdown Induced Cell Cycle Arrest and Apoptosis

The role of TROAP in regulating the cell cycle and apoptosis was evaluated by flow cytometry. Based on our cell cycle analysis, we found that TROAP knockdown induced S phase arrest (**Figures 2I, J**, 47.72% in shTROAP *vs* 41.81% in shCtrl, *P <* 0.01). Further, the apoptosis analysis showed that TROAP knockdown promoted cell apoptosis (**Figures 2K, L**, 7.25% in shTROAP *vs* 2.88% in shCtrl, *P <* 0.01).

### TROAP Knockdown Inhibited Migration and Invasion Abilities of Prostate Cancer Cells

**Figure 3:**
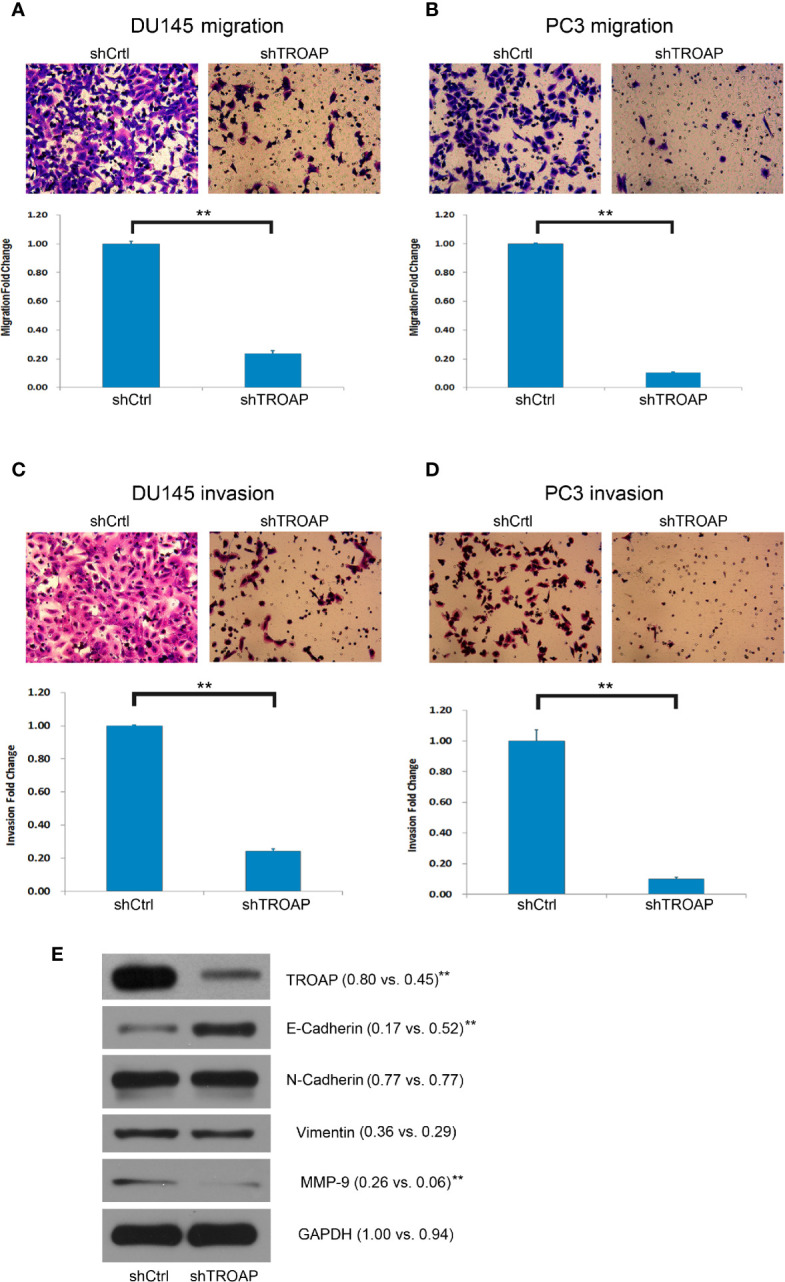
Cell invasion and migration assays in DU145 and PC-3 cells. Cell migration assay results indicate that TROAP depletion in DU145 and PC3 cells significantly suppressed the migration ability of the prostate cancer cells **(A, B)**. The invasion assay results were similar with low migration of the cells **(C, D)**. TROAP knockdown inhibited expression of MMP-9 and promoted expression of E-Cad **(E)**. Data are presented as the mean ± standard deviation. **P < 0.01.

Aberrant cell invasion and migration are frequently correlated with prostate cancer, and enhanced invasion and migration abilities are thought to be correlated with cancer metastases. Therefore, we assessed the biological role of TROAP in DU145 and PC-3 cells using Transwell assays. Our results showed that TROAP depletion significantly suppressed the migration of DU145 and PC-3 cells by 76.33% and 89.62%, respectively (**Figures 3A, B**, *P <* 0.01). Moreover, the Transwell invasion assay results showed that TROAP knockdown suppressed the invasion abilities of DU145 and PC-3 cells by 75.70% and 89.92%, respectively (**Figures 3C, D**, *P <* 0.01). Furthermore, we quantified the expression of EMT (epithelial-mesenchymal transition)-related proteins in PC-3 cells and found that TROAP knockdown inhibited the expression of MMP-9 and promoted the expression of E-Cadherin (**Figure 3E**).

### TROAP Silencing Inhibited the TWIST/c-Myc Pathway

**Figure 4:**
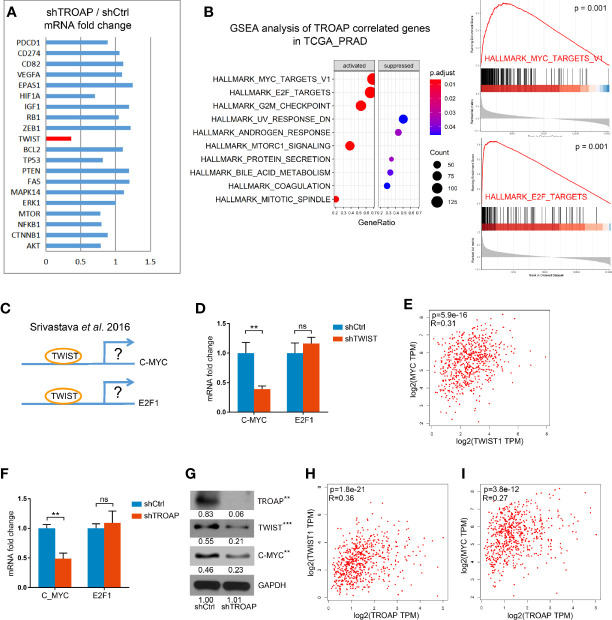
qPCR results show that TROAP knockdown inhibited TWIST expression **(A)**. GSEA analysis of TROAP coexpressed genes showed that c-Myc and E2F1 signals were correlated with TROAP expression **(B)**. TWIST could bound to the promoter regions of E2F1 and c-Myc to regulate E2F1 and c-Myc expression **(C)**. TWIST knockdown could inhibit the expression of c-Myc and had no effect on expression of E2F1 **(D)**. Analysis of the TCGA database showed a correlation between TWIST and c-Myc **(E)**. TROAP knockdown suppressed TWIST and c-Myc expressions at the levels of mRNA and protein **(F, G)**. TROAP expression was positively correlated with TWIST and c-Myc expressions **(H, I)**. **P < 0.01, ***P < 0.001. ns means no significance.

To identify the potential downstream genes of TROAP we selected 20 genes associated with cancer cell cycles, EMT, or apoptosis and quantified their expression with qPCR in shTROAP and shCtrl prostate cancer cells. Our results showed that expression of TWIST was significantly down-regulated in the TROAP-silenced cells (**Figure 4A**, 36.84%, *P <* 0.001). A GSEA analysis of genes coexpressed with TROAP showed that c-Myc and E2F1 signals had a strong correlation with TROAP (**Figure 4B**). We inferred that TROAP might interact with c-Myc, E2F1, and TWIST in prostate cancer cells, but the association among the four genes was unclear.

A previous study showed that TWIST could bind to the promoter regions of E2F1 and c-Myc to regulate E2F1 and c-Myc expression (**Figure 4C**) (13). Thus, we quantified the expressions of E2F1 and c-Myc mRNAs in cells infected with shTWIST lentivirus (shTWIST lentivirus inhibited TWIST mRNA expression by 81.55%, not shown). These results indicate that knockdown of TWIST could inhibit the expression of c-Myc by 59.65% and that it had no effect on the expression of E2F1 (**Figure 4D**). Further verification in the TCGA database showed a strong correlation between TWIST and c-Myc (**Figure 4E**).

To explore the role of TROAP in the regulation of TWIST, E2F1, and c-Myc, we performed qPCRs and western blots. Our results showed that TROAP knockdown suppressed the expression of TWIST and c-Myc at the mRNA and protein levels (**Figures 4F, G**). Also, the coexpression analysis showed that TROAP expression was positively correlated with those of TWIST and c-Myc (**Figures 4H, I**). These results indicate that TROAP could regulate c-Myc expression *via* TWIST.

### TWIST Overexpression Partially Abrogated the Inhibitory Effects of the TROAP Knockdown on Prostate Cancer Cells

**Figure 5:**
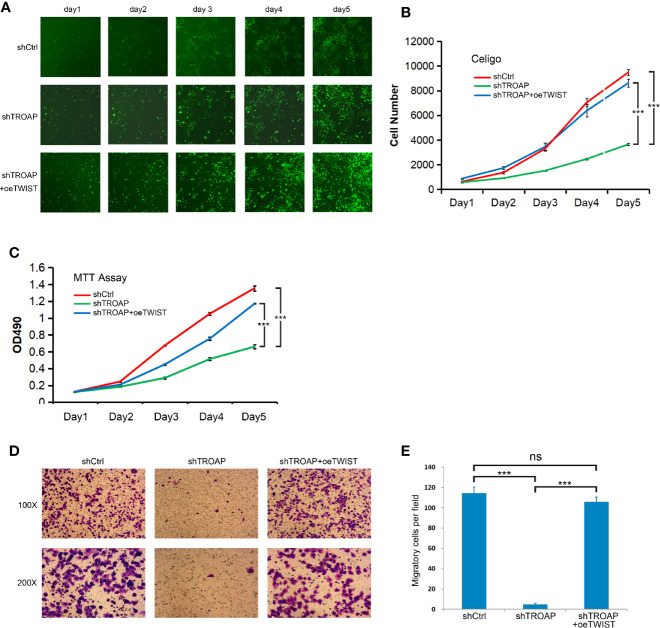
TWIST upregulation partially abrogated the inhibition mediated by TROAP knockdown on prostate cancer cells **(A, B)**. Overexpression partially abrogated the inhibition of the cell proliferation induced by TROAP silencing. Overexpression of TWIST reversed the inhibitory effect on cell viability **(C)** and cell invasion **(D, E)**. ***P < 0.001; ns, no significant.

Following the determination that TWIST is a downstream target of TROAP, we performed additional experiments to explore whether TWIST mediates the inhibitory effect of TROAP knockdown. We constructed a TWIST overexpression lentivirus (oeTWIST) and coinfected prostate cancer cells with oeTWIST and shTWIST lentiviruses, or with negative control; next, we performed cell proliferation, viability, and invasion assays. According to the cell numbers calculated with Celigo cell imaging analyzer, TWIST overexpression partially abrogated the inhibition of the cell proliferation induced by TROAP silencing (**Figures 5A, B**). Similarly, TWIST overexpression partially reversed the inhibitory effect on cell viability (**Figure 5C**, MTT) and cell invasion ability (**Figures 5D, E**, Transwell invasion assay). All these results demonstrate that TWIST upregulation partially abrogated the inhibition of the effect mediated by TROAP knockdown on prostate cancer cells, which further suggests that TWIST might be a major downstream target regulated by TROAP.

### TROAP and E2F1 Are Co-Regulated by EZH2

**Figure 6:**
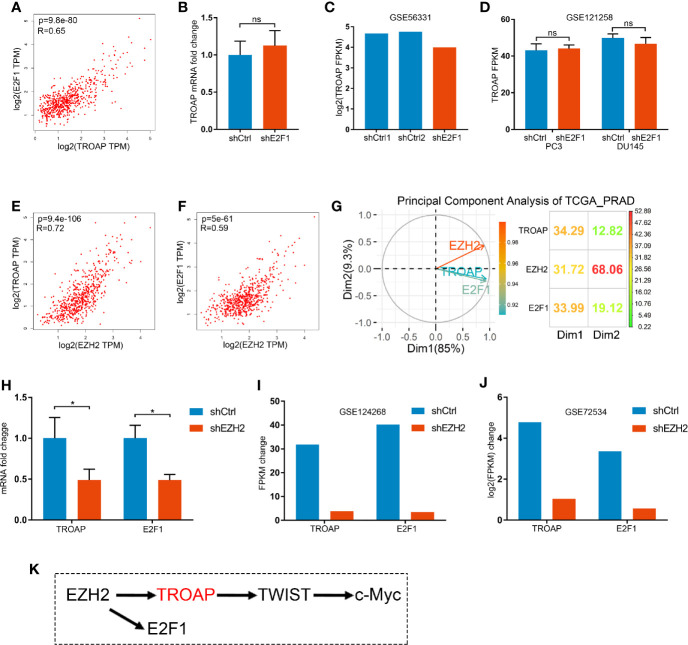
Correlation between the expressions of E2F1 and TROAP **(A)**. Knockdown of E2F1 produced no effect on the expression of TROAP **(B–D)**. Expression of EZH2 was correlated with TROAP and E2F1 expressions **(E, F)**. Results of the principal component analysis (PCA) show that EZH2 was a driver in prostate cancer **(G)**. qPCR **(H)** and microarray **(I, J)** results confirm that knockdown of EZH2 inhibited the expressions of E2F1 and TROAP. TROAP and E2F1 are regulated by EZH2, and TROAP induced the TWIST/c-Myc pathway to regulate the prostate cancer cell process **(K)**. *P < 0.05; ns, no significant.

The interaction between E2F1 and TROAP was unclear. Our coexpression analysis showed a strong correlation between E2F1 and TROAP (**Figure 6A**). However, qPCR (**Figure 6B**) and microarray (**Figures 6C, D**) results showed that E2F1 knockdown had no effect on the expression of TROAP. Thus, E2F1 and TROAP cannot regulate each other directly (based on **Figures 4F**, **6B–D**). In our previous study (14), we had noticed that E2F1 and TROAP were coexpressed with EZH2; therefore, we further explored the expressions of EZH2 and those two genes. Similarly to our previous results, we found a strong correlation between the expression of EZH2 and those of either TROAP or E2F1 (**Figures 6E, F**). To determine which of the three genes plays the key role in prostate cancer, we performed a principal component analysis (PCA) using the TCGA_PRAD and the results showed that EZH2 plays a more important driving role in prostate cancer (**Figure 6G**). Our qPCR (**Figure 6H**) and microarray (**Figures 6I, J**) results confirmed that EZH2 knockdown inhibited the expressions of E2F1 and TROAP. And knockdown of E2F1 could inhibit expression of EZH2 only in PC3 cells (**Supplementary Figure 1**). Thus, TROAP and E2F1 seem to be coregulated by EZH2 (**Figure 6K**).

## Discussion

TROAP is considered a critical cell adhesion molecule for the function of trophinin (14). TROAP plays important roles in cellular processes; it acts as a part of the adhesion complex and plays an essential role during early embryo implantation (5, 15). In addition, TROAP is thought to be essential for cell proliferation, because it was shown to be required for spindle assembly and centrosome integrity during mitosis (16). At the same time, TROAP was shown to be abnormally expressed in some tumor tissues and cell lines (17–19), including in prostate cancer (12), and it could regulate cancer cell biological processes. Nonetheless, little is known about the role of TROAP in prostate cancer development and progression.

In the present study, we analyzed TROAP expression in TCGA and GEO databases. The results showed that TROAP expression was obviously higher in prostate cancer than in normal prostate tissues, and that it was also higher in prostate cancers with higher Gleason scores, higher T or N stages, and in recurrent cancers than in other prostate cancers. We further investigated the potential biological functions of TROAP *in vitro*. Our results showed that TROAP knockdown inhibited cell proliferation and induced cell apoptosis by mediating cell cycle arrest on the S phase. We also observed that cell apoptosis rate rises not significantly (7.25% in shTROAP vs 2.88% in shCtrl). The inhibition of cell proliferation might be caused mainly by the cell cycle arrest on the S phase. The S phase of cell cycle allows cell DNA replication without accumulating genetic abnormalities. Some anti-tumor drugs, such as hinokitiol and xanthohumol, arrest cells in an S phase and inhibit cell growth (20, 21). In our study, TROAP showed a growth inhibitory effect by arresting cells in the S phase of the cycle.

TWIST is a transcription factor shown to mediate activation of EMT in multiple cancer cells (22, 23). Our results showed that TROAP knockdown could suppress prostate cancer cell migration and invasion by inhibiting TWIST and MMP-9 expressions. To further confirm the role of TROAP in the gene regulatory network in prostate cancer, we assessed gene coexpression by qPCR. Our results indicate that TROAP and E2F1 were co-regulated by EZH2 and that TROAP could regulate cell processes partially *via* the TWIST/c-Myc pathway. In addition, TWIST and c-Myc could both promote tumor development. However, the mechanisms by which TWIST interacts with c-Myc are differently described in different studies (13, 24). The c-Myc protein can bind and activate the TWIST1 promoter to promote the expression of TWIST1 (24). But, TWIST can also bind the promoter regions of c-Myc at the canonical E-box binding motif and directly regulate its transcription (13). During the development of different tumors, different pathways or transcription factors play different leading roles at different stages, and this may explain the changing regulatory roles between TWIST and c-Myc. In prostate cancer, TWIST knockdown could suppress the expression of c-Myc.

In this study, we used PCA to distinguish the main factor among EZH2, TROAP and E2F1 causing tumorigenesis and development of prostate cancer. PCA is a method in dimension reduction, with numerous applications in biology, chemistry, and many other disciplines (25, 26). It is an efficient method capable of identifying independent eigenmodes responsible for observed variations (26, 27). The results of our PCA showed that the three genes are important for prostate cancer, but that EZH2 probably plays a driving role.

In summary, we observed overexpression of TROAP in prostate cancer when compared with the expression in normal tissues. Our experiment results indicate that TROAP could promote prostate cancer development and progression, at least partially, *via* a TWIST/c-Myc pathway. Furthermore, our observations suggest that TROAP and E2F1 are co-regulated by EZH2. Taken together, these findings suggest novel prostate cancer treatment targets.

## Data Availability Statement

The raw data supporting the conclusions of this article will be made available by the authors, without undue reservation.

## Author Contributions

All authors contributed to data analysis, drafting, or revising the article. All authors contributed to the article and approved the submitted version.

## Funding

This study was supported by grants for JZ from the National Natural Science Foundation of China (no. 81773221), the Natural Science Foundation of Jiangsu Province (no. BK20161222), the Suzhou Science and Technology Planed Projects (no. SS201857), and the Key Young Talents of Medicine in Jiangsu (no.QNRC2016875). Grants for LJ from CNNC Science Fund for Talented Young Scholars (2018-272-4).

## Conflict of Interest

The authors declare that the research was conducted in the absence of any commercial or financial relationships that could be construed as a potential conflict of interest.

## Supplementary Material

Supplementary Figure 1 Knockdown of E2F1 inhibited expression of EZH1 in PC3 cells. In DU145 cells it is not obvious.
