# Supplementary figures and images for: EZH2-TROAP Pathway Promotes Prostate Cancer Progression Via TWIST Signals
Source: Front Oncol. 2021 Feb 22;10:592239. doi: 10.3389/fonc.2020.592239 (PMC7938320; doi:10.3389/fonc.2020.592239)

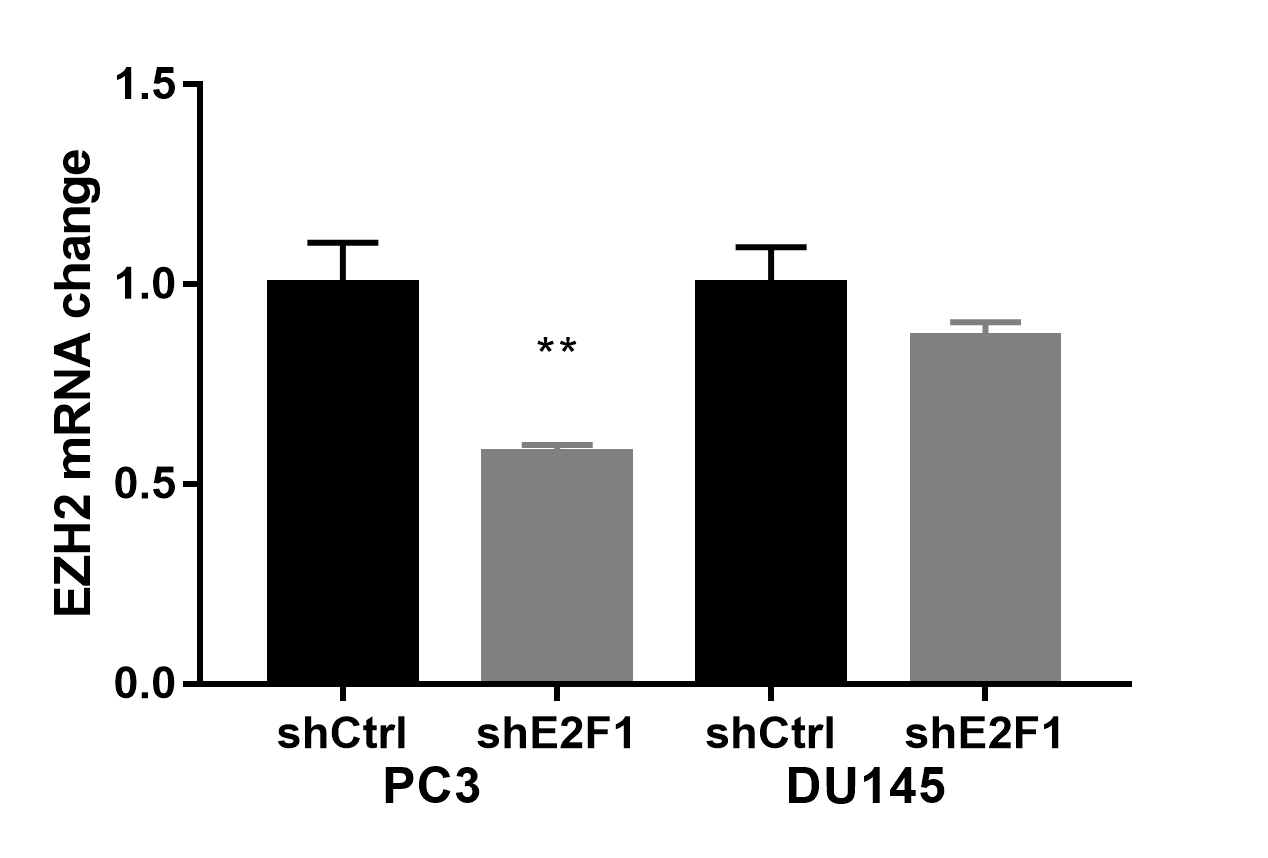

Supplement: Supplementary Figure 1 — Knockdown of E2F1 inhibited expression of EZH1 in PC3 cells. In DU145 cells it is not obvious. [file Image_1.tif]
