# Supplementary material for: EZH2-TROAP Pathway Promotes Prostate Cancer Progression Via TWIST Signals
Source: Front Oncol. 2021 Feb 22;10:592239. doi: 10.3389/fonc.2020.592239 (PMC7938320; doi:10.3389/fonc.2020.592239)
Supplement: Supplementary file 2 [file Table_1.docx]

| **Name of Antibody** | **Biological Source** | **Company** | **Item Number** |
| --- | --- | --- | --- |
| Snail | Rabbit | CST | #3879 |
| AKT | Rabbit | abcam | ab179463 |
| β-Catenin | Rabbit | CST | #8480 |
| Slug | Rabbit | CST | #9585 |
| Vimentin | Rabbit | CST | #3932 |
| P-β-Catenin | Rabbit | CST | #2009 |
| GAPDH | Rabbit | abcam | ab37168 |
| TROAP | Rabbit | Sigma | HPA044102 |

Supplementary Table 1. Antibodies used in the present study.
